# *P3N-PIPO* but not *P3* is the avirulence determinant in melon carrying the *Wmr* resistance against watermelon mosaic virus, although they contain a common genetic determinant

**DOI:** 10.1128/jvi.00507-24

**Published:** 2024-05-22

**Authors:** Zhen Sun, Yu-Xuan Wu, Ling-Zhi Liu, Yan-Ping Tian, Xiang-Dong Li, Chao Geng

**Affiliations:** 1 Department of Plant Pathology, Shandong Provincial Key Laboratory of Agricultural Microbiology, College of Plant Protection, Shandong Agricultural University, Tai’an, Shandong, China; 2 Institute of Plant Protection, Shandong Academy of Agricultural Sciences, Ji’nan, Shandong, China; Emory University School of Medicine, Atlanta, Georgia, USA

**Keywords:** P3N-PIPO, avirulence determinant, *Wmr *resistance, transcriptional slippage, *Potyvirus*, watermelon mosaic virus

## Abstract

Viruses employ a series of diverse translational strategies to expand their coding capacity, which produces viral proteins with common domains and entangles virus-host interactions. P3N-PIPO, which is a transcriptional slippage product from the *P3* cistron, is a potyviral protein dedicated to intercellular movement. Here, we show that P3N-PIPO from watermelon mosaic virus (WMV) triggers cell death when transiently expressed in *Cucumis melo* accession PI 414723 carrying the *Wmr* resistance gene. Surprisingly, expression of the P3N domain, shared by both P3N-PIPO and P3, can alone induce cell death, whereas expression of P3 fails to activate cell death in PI 414723. Confocal microscopy analysis revealed that P3N-PIPO targets plasmodesmata (PD) and P3N associates with PD, while P3 localizes in endoplasmic reticulum in melon cells. We also found that mutations in residues L35, L38, P41, and I43 of the P3N domain individually disrupt the cell death induced by P3N-PIPO, but do not affect the PD localization of P3N-PIPO. Furthermore, WMV mutants with L35A or I43A can systemically infect PI 414723 plants. These key residues guide us to discover some WMV isolates potentially breaking the *Wmr* resistance. Through searching the NCBI database, we discovered some WMV isolates with variations in these key sites, and one naturally occurring I43V variation enables WMV to systemically infect PI 414723 plants. Taken together, these results demonstrate that P3N-PIPO, but not P3, is the avirulence determinant recognized by Wmr, although the shared N terminal P3N domain can alone trigger cell death.

**IMPORTANCE:**

This work reveals a novel viral avirulence (Avr) gene recognized by a resistance (R) gene. This novel viral Avr gene is special because it is a transcriptional slippage product from another virus gene, which means that their encoding proteins share the common N-terminal domain but have distinct C-terminal domains. Amazingly, we found that it is the common N-terminal domain that determines the Avr-R recognition, but only one of the viral proteins can be recognized by the R protein to induce cell death. Next, we found that these two viral proteins target different subcellular compartments. In addition, we discovered some virus isolates with variations in the common N-terminal domain and one naturally occurring variation that enables the virus to overcome the resistance. These results show how viral proteins with common domains interact with a host resistance protein and provide new evidence for the arms race between plants and viruses.

## INTRODUCTION

Plant viruses cause an estimated economic loss of approximately 30 billion dollars every year, posing a significant challenge to agriculture worldwide (1). Planting resistant cultivars is the most effective way to control plant viruses. Resistant cultivars usually carry dominant or recessive resistance (R) genes. According to the gene-for-gene model, R genes specifically recognize the avirulence (Avr) genes from pathogens (2, 3). Some R genes encode nucleic-binding domain leucine-rich repeat-containing receptors (NLRs), which directly or indirectly recognize viral effectors to induce effector-triggered immunity, culminating in rapidly programmed cell death referred to as hypersensitive response (4, 5). Research on Avr genes provides an understanding of virus-host incompatible interaction and co-evolution of viruses and plants.

Identification of actual viral Avr effectors recognized by R proteins is challenged when involved in viral proteins with common domains. These viral proteins are usually encoded by plant viruses adopting noncanonical translational strategies, such as ribosomal frameshift, ribosomal readthrough, and transcriptional slippage (6–8). Ribosomal frameshifting and readthrough can change the reading frame of the viral genome template to produce two viral proteins with common and different domains (9), whereas transcriptional slippage enables the virus to encode two viral proteins with common and distinct domains by adding nucleotides in viral genomes (10, 11). Transcriptional slippage has been found in plant viruses from the family Potyviridae (6, 12), which comprises the largest group of plant-infecting RNA viruses.

*Potyvirus* is the largest genus in the family Potyviridae, which includes many economically important viruses, such as potato virus Y, plum pox virus, turnip mosaic virus (TuMV), soybean mosaic virus (SMV), watermelon mosaic virus (WMV, formerly called WMV II), etc. (13–16). The genome of potyviruses is positive-sense single-stranded RNA that encodes two polyproteins, which are cleaved into 10–12 mature proteins by P1, HC-Pro, and NIa-Pro (17). Among them, *P3N-PIPO* is expressed by transcriptional slippage in the open reading frame (ORF) of *P3* (6, 12). There is a “G_1-2_A_6-7_” motif in the middle region of *P3* (12). The slippage of the potyviral polymerase NIb in this region leads to a part of transcript with an extra nucleotide “A,” which changes the translation order of subsequent amino acids to express Pretty Interesting Potyviridae ORF (PIPO) (6, 18). This PIPO domain is expressed as a fusion with the P3N domain (19, 20). Therefore, both P3 and P3N-PIPO share the common P3N domain, while the C-terminal region between P3N-PIPO and P3 is distinct. P3 not only functions in viral genome replication but also acts as a pathogenicity and symptom determinant in certain potyviruses (21, 22). In contrast, P3N-PIPO participates in the intercellular movement of potyviruses (19, 20, 23, 24). It targets plasmodesmata (PD) and recruits cylindrical inclusions (CIs) from the cytoplasm to PD via the PIPO domain (23). P3N-PIPO can interact with the host protein PCaP1 or DREPP at PD (19, 20). P3N-PIPO also interacts with P3 via the shared P3N domain to recruit viral replication vesicles for intercellular movement (21). Apart from contributing to the cell-to-cell movement of potyviruses, P3N-PIPO also participates in virus virulence and resistance recognition. For example, the clover yellow vein virus (ClYVV) isolate 90-1 Br2 can break the *cyv1*-mediated recessive resistance, mainly through accumulating a higher amount of P3N-PIPO than the nonbreaking isolate ClYVV isolate Cl-no30 (25). In addition, P3N-PIPO not only pleiotropically determines the virulence of ClYVV in susceptible peas (26), but also contributes to the synergism between ClYVV and white clover mosaic virus (WClMV) in peas (27). However, the role of P3N-PIPO in triggering a resistance response remains largely unclear.

WMV, one of the economically important potyviruses, is very prevalent in melon and zucchini crops (28). Planting resistant cultivars is an effective way to control WMV. A previous study showed *Cucumis melo* accession PI 414723 that carries a single dominant resistance gene *Wmr* is resistant to WMV isolate NY 62-76 (29). However, the potential viral protein recognized by Wmr remains unknown. In this study, we reveal WMV P3N-PIPO, but not P3, triggers cell death in PI 414723 plants, although the P3N domain shared by P3N-PIPO and P3 can induce cell death. We further show both P3N-PIPO, which targets PD, and P3N, which associates with PD, cause cell death, while P3 which localizes in endoplasmic reticulum (ER) fails to trigger cell death. In addition, mutations in the amino acid residues L35 or I43 of the P3N region disrupt the P3N-PIPO-triggered cell death and enable WMV to overcome host resistance in PI 414723 plants. These mapped residues contribute to finding WMV isolates that potentially break the *Wmr* resistance, which is meaningful for the reasonable layout of disease-resistant varieties. The new data enlarge the knowledge of interactions between resistance proteins and viral proteins.

## RESULTS

### *C. melo* accession PI 414723 with the *Wmr* resistance gene is resistant to WMV

**Fig 1:**
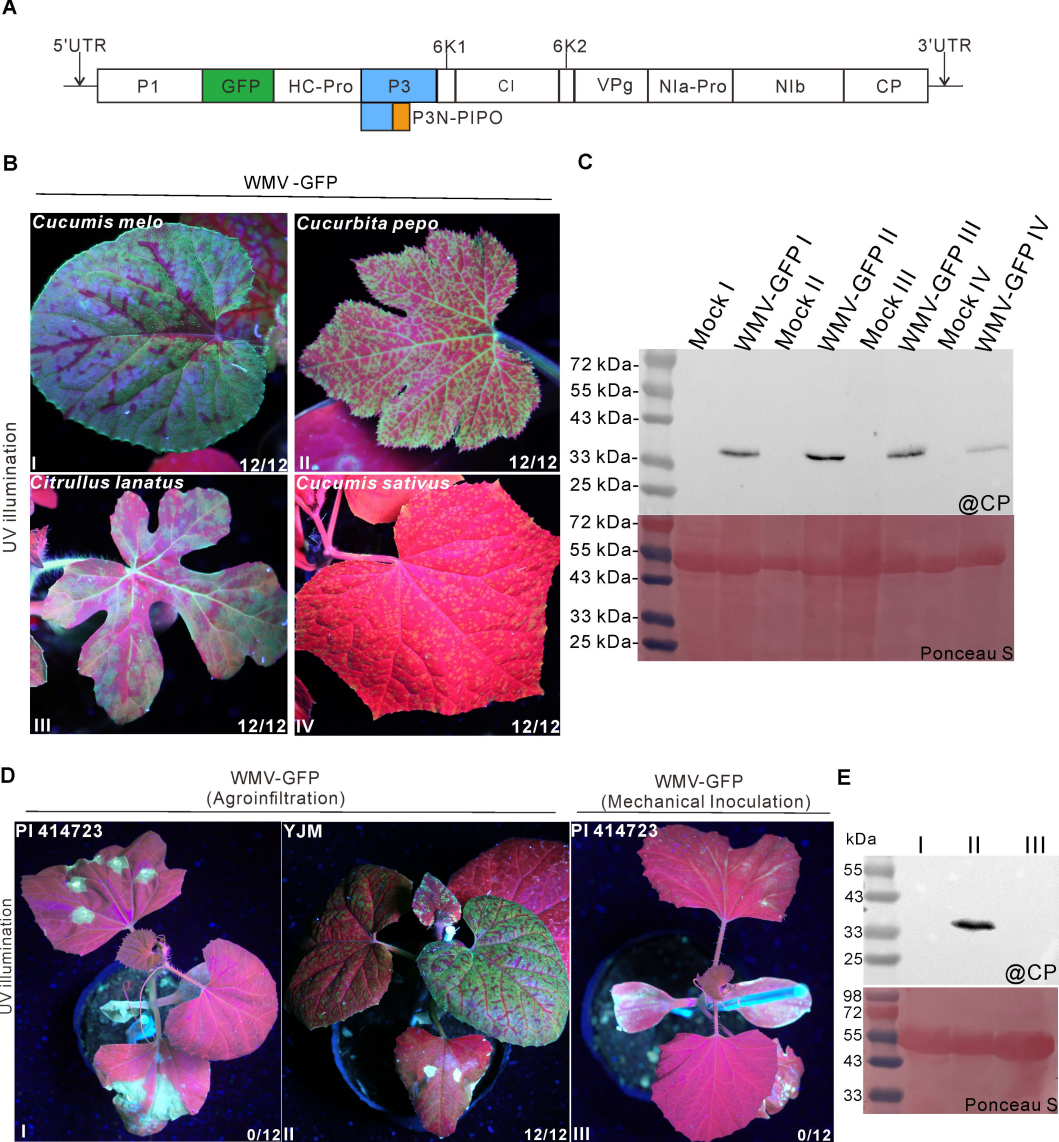
*Cucumis melo* accession PI 414723 is resistant to WMV. (**A**) A schematic diagram of WMV-GFP. A *GFP* tag was inserted between *P1* and *HC-Pro*. (**B**) Phenotypes of *Cucumis melo*, *Cucurbita pepo*, *Citrullus lanatus*, and *Cucumis sativus* plants individually infected with WMV-GFP under the ultraviolet (UV) illumination at 10 days post-agroinfiltration (dpai). The fraction represents the number of plants exhibiting fluorescence out of 12 inoculated plants from three independent experiments. (**C**) Western blot analysis of virus accumulation in the upper leaf tissues of plants inoculated with WMV-GFP. Total proteins were extracted from systemically infected leaves and detected using the WMV CP antibody. Ponceau S staining indicates the loading control. (**D**) Phenotypes of PI 414723 and YJM plants inoculated WMV-GFP at 15 dpai or 15 dpmi, respectively. (**E**) Immunoblot analysis of virus accumulation in the upper leaf tissues of plants inoculated with WMV-GFP. Ponceau S staining shows protein loading of samples.

To investigate the incompatible interactions between WMV and PI 414723 harboring the *Wmr* resistance gene, we constructed a cDNA clone of WMV isolate GN collected from *Cucurbita pepo* in Shandong Province, China. A *GFP* tag was inserted between *P1* and *HC-Pro* (Fig. 1A). The resultant cDNA clone pCB301-WMV-GFP was inoculated into different cucurbit plants through *Agrobacterium*-mediated infiltration (agroinfiltration), respectively. After 10 days post-agroinfiltration (dpai), green fluorescence was individually observed in the upper uninoculated leaves of *Cucumis melo*, *Cucurbita pepo*, *Citrullus lanatus*, and *Cucumis sativus* plants (Fig. 1B). Western blot analysis showed the coat protein (CP) of WMV accumulated in the corresponding plant tissues (Fig. 1C), suggesting the cDNA clone pCB301-WMV-GFP is infectious. We then inoculated this infectious clone into PI 414723 and *C. melo* cultivar Yangjiaomi (YJM) plants by agroinfiltration, respectively. At 15 dpai, cell death appeared in the inoculated leaves of PI 414723, but did not in that of YJM (Fig. 1D). Furthermore, green fluorescence distributed in the upper uninoculated leaves of YJM, but did not in that of PI 414723 (Fig. 1D). Immunoblot analysis showed WMV CP only accumulated in the upper uninoculated leaves of YJM, but did not in that of PI 414723 (Fig. 1E). A previous study showed PI 414723 plants infected with WMV isolate NY 62-76 initially displayed mosaic symptoms in inoculated leaves, but eventually recovered from symptoms (29). Moreover, low or no virus was detected in the youngest leaves in the enzyme-linked immunosorbent assay (29). To verify the PI 414723 resistance against WMV isolate GN, virus particles were extracted and purified from YJM plants infected with WMV-GFP and then rubbed onto the surface of PI 414723 leaves. At 15 days post-mechanical inoculation (dpmi), a few necrotic spots were observed in the inoculated leaves of PI 414723 plants, and no green fluorescence was observed in the upper uninoculated leaves (Fig. 1D). Western blot analysis showed WMV CP was not detected in PI 414723 plants (Fig. 1E). Therefore, the PI 414723 resistance against WMV may vary in response to different WMV isolates. Together, these results reveal that PI 414723 is resistant to WMV.

### Both P3N-PIPO targeting PD and P3N associating with PD can trigger cell death

**Fig 2:**
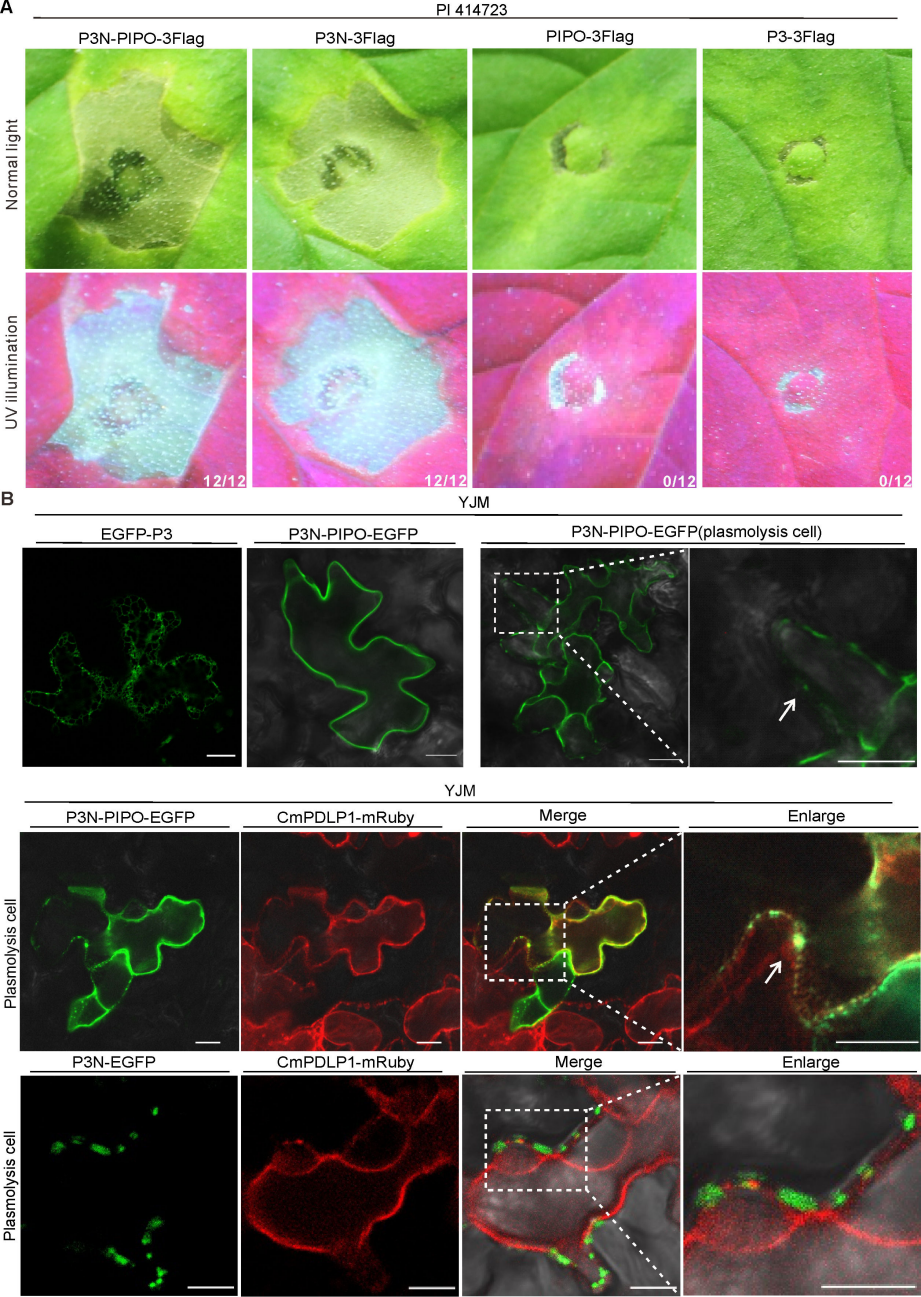
Both P3N-PIPO targeting PD and P3N associating with PD trigger cell death. (**A**) Phenotypes of PI 414723 individually expressing P3N-PIPO-3Flag, P3N-3Flag, PIPO-3Flag, and P3-3Flag under normal light or UV illumination at 3 dpai, respectively. The fraction represents the number of leaf patches showing cell death out of 12 agroinfiltrated patches from three independent experiments. (**B**) Subcellular localization analysis of EGFP-P3, P3N-PIPO-EGFP, and P3N-EGFP in YJM leaves at 3 dpai, respectively. CmPDLP1 is a PD marker. The boxes indicate the enlarged area. The arrows point to the co-localized signal at PD. Scale bar, 10 µm.

To identify the WMV protein responsible for inducing cell death in PI 414723, we transiently expressed N-terminally or C-terminally 3 × Flag (3Flag hereafter)-tagged WMV proteins in PI 414723 and YJM leaves, respectively. At 3 dpai, cell death only appeared in the leaf patches expressing P3N-PIPO-3Flag in PI 414723 (Fig. 2A; Fig. S1A and B). In contrast, transient expression of P3N-PIPO did not trigger cell death in YJM (Fig. S1C). Western blot analysis showed P3N-PIPO was expressed properly in YJM (Fig. S1D). These results suggest that P3N-PIPO is the WMV protein responsible for activating cell death in PI 414723.

To identify the domain crucial for P3N-PIPO-triggered cell death, we transiently expressed the P3N and PIPO domains in PI 414723 leaves, respectively. Results showed the leaf patches expressing the P3N domain displayed cell death at 3 dpai, while those expressing the PIPO domain did not (Fig. 2A). Immunoblot analysis confirmed both the P3N domain and PIPO domain were expressed properly (Fig. S1E). Because the P3N domain is shared by both P3 and P3N-PIPO (Fig. 1A), we tested whether P3 activated cell death. Surprisingly, we found transient expression of N-terminally or C-terminally 3Flag-tagged P3 failed to induce cell death in PI 414723 leaves (Fig. 2A; Fig. S1F). To characterize why P3 does not cause cell death, we compared the subcellular localization of P3 and P3N-PIPO in YJM leaf cells. Confocal microscopy analysis showed EGFP-P3 was located in the endoplasmic reticulum, while P3N-PIPO-EGFP colocalized with the PD marker CmPDLP1-mRuby (Fig. 2B). We also analyzed the subcellular localization of the P3N domain. Results showed that P3N-EGFP was associated with CmPDLP1-mRuby at the cell wall (Fig. 2B). These results suggest that both P3N-PIPO, which targets PD, and P3N, which associates with PD, induce cell death in PI 414723.

### Mutations in residues L35, L38, P41, and I43 of the P3N domain individually compromise cell death induced by P3N-PIPO

**Fig 3:**
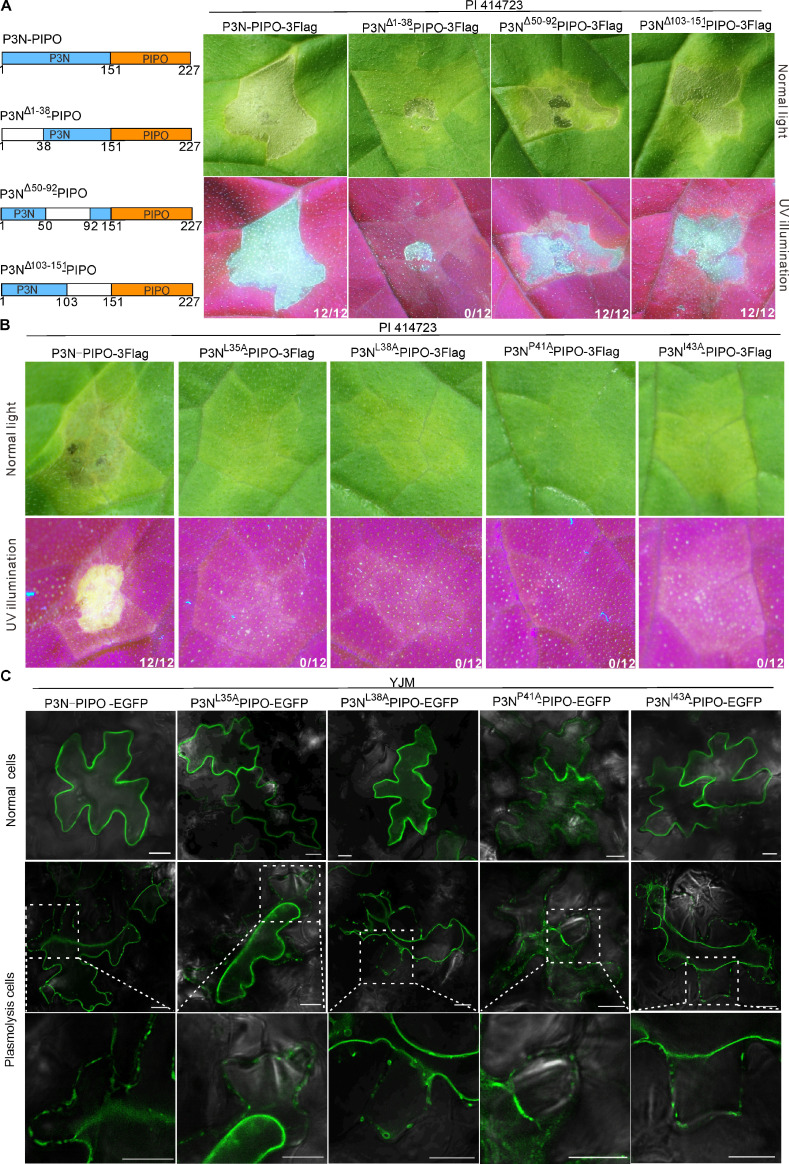
Mutations in residues L35, L38, P41, and I43 of the P3N domain individually compromise P3N-PIPO-induced cell death. (**A**) Phenotypes of PI 414723 expressing P3N-PIPO and P3N-PIPO truncated mutants under normal light or UV illumination at 3 dpai, respectively. The schematic diagram gives detailed information about P3N-PIPO truncated mutants, in which P3N^Δ1-38^-PIPO was truncated from the N-terminal region [1-38 amino acid (AA) residue] of P3N, P3N^Δ50-92^-PIPO was truncated from the middle region (50-92 AA residues) of P3N, and P3N^Δ103-151^-PIPO was truncated from the C-terminal region (103-151 AA residues) of P3N. (**B**) Phenotypes of PI 414723 leaves expressing P3N-PIPO-3Flag, P3N-PIPO^L35A^-3Flag, P3N-PIPO^L38A^-3Flag, P3N-PIPO^P41A^-3Flag, and P3N-PIPO^I43A^-3Flag under normal light or UV illumination at 3 dpai, respectively. For panels (A) and (B), the fraction represents the number of leaf patches exhibiting cell death out of 12 agroinfiltrated patches from three independent experiments. (**C**) Subcellular localization analysis of P3N-PIPO and P3N-PIPO mutants in YJM leaves at 3 dpai, respectively. Scale bar, 10 µm.

To further investigate the key region determining to trigger cell death in the P3N domain, we constructed three P3N-truncated mutants, namely P3N^Δ1-38^-PIPO, P3N^Δ50-92^-PIPO, and P3N^Δ103-151^-PIPO (Fig. 3A). When transiently expressed in PI 414723 leaves, P3N^Δ1-38^-PIPO failed to cause cell death in the infiltrated leaf patches, while P3N^Δ50-92^-PIPO and P3N^Δ103-151^-PIPO still triggered cell death as wild-type P3N-PIPO did (Fig. 3A). Western blot analysis showed three P3N-truncated mutants were expressed properly in leaves (Fig. S1G). Thus, the amino acid residues from 1 to 38 in the N-terminus of the P3N domain are required for P3N-PIPO-induced cell death in PI 414723.

We next performed alanine scanning in the P3N^1-38^ region. The resultant mutants were individually expressed in PI 414723 leaves. At 3 dpai, cell death was not observed in the leaf patches expressing P3N^L35A^-PIPO-3Flag, P3N^L38A^-PIPO-3Flag, P3N^P41A^-PIPO-3Flag, and P3N^I43A^-PIPO-3Flag, respectively (Fig. 3B). Immunoblot analysis showed that all mutants were expressed properly in leaves (Fig. S1H). We also analyzed the subcellular localization of these mutants. The C-terminally EGFP-tagged P3N-PIPO mutants that failed to trigger cell death were transiently expressed in YJM cells. Results showed P3N^L35A^-PIPO-EGFP, P3N^L38A^-PIPO-EGFP, and P3N^I43A^-PIPO-EGFP displayed a similar subcellular localization pattern with wild-type P3N-PIPO-EGFP, whereas P3N^P41A^-PIPO-EGFP accumulated in the cytoplasm in addition to targeting PD (Fig. 3C). Together, the residues L35, L38, P41, and I43 in the P3N domain of P3N-PIPO are key to P3N-PIPO-induced cell death.

### WMV mutants with the L35A or I43A mutation can systemically infect PI 414723

**Fig 4:**
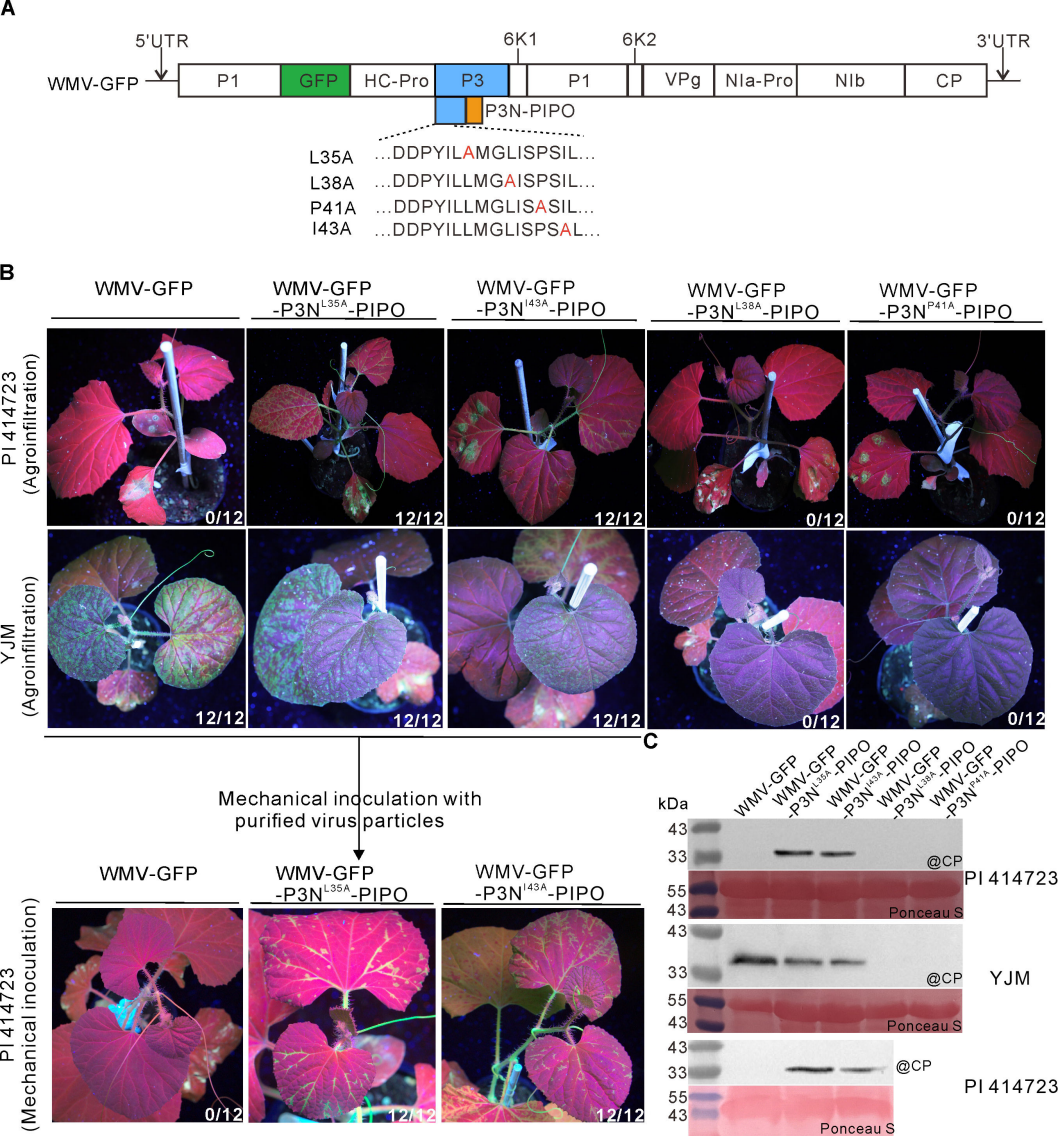
Mutations in the amino acid residue L35 or I43 of the P3N domain enable WMV to systemically infect PI 414723. (**A**) Schematic diagrams of the mutants WMV-GFP-P3N^L35A^-PIPO, WMV-GFP-P3N^L38A^-PIPO, WMV-GFP-P3N^P41A^-PIPO, and WMV-GFP-P3N^I43A^-PIPO. (**B**) Phenotypes of *C. melo* plants individually inoculated with WMV-GFP, WMV-GFP-P3N^L35A^-PIPO, WMV-GFP-P3N^I43A^-PIPO, WMV-GFP-P3N^L38A^-PIPO, and WMV-GFP-P3N^P41A^-PIPO under the UV illumination. The first panel displays the phenotypes of PI 414723 plants infiltrated with *Agrobacterium* carrying the indicated infectious clone at 10 dpai. The second panel exhibits the phenotypes of YJM plants infiltrated with *Agrobacterium* carrying the indicated infectious clone at 20 dpai. The third panel shows the phenotypes of YJM plants mechanically inoculated with purified virus particles extracted from WMV-GFP-P3N^L35A^-PIPO or WMV-GFP-P3N^I43A^-PIPO infected PI 414723 plants at 20 dpmi. The fraction represents the number of inoculated plants exhibiting fluorescence in the upper uninoculated leaves out of 12 agroinfiltrated plants from three independent experiments. (**C**) Western blot analysis of the coat protein accumulation of the upper uninoculated leaves in panel (B). Ponceau S staining shows protein loading of samples.

To test whether the above mutations compromising cell death enable WMV to break the *Wmr* resistance, the equivalent mutations were individually introduced into the infectious clone pCB301-WMV-GFP (Fig. 4A). The resultant mutants WMV-GFP-P3N^L35A^-PIPO, WMV-GFP-P3N^L38A^-PIPO, WMV-GFP-P3N^P41A^-PIPO, and WMV-GFP-P3N^I43A^-PIPO were inoculated into PI 414723 plants by agroinfiltration, respectively. At 10 dpai, green fluorescence was observed in both the inoculated and upper uninoculated leaves of WMV-GFP-P3N^L35A^-PIPO and WMV-GFP-P3N^I43A^-PIPO infected PI 414723 plants, but only in the inoculated leaves of WMV-GFP-P3N^L38A^-PIPO and WMV-GFP-P3N^P41A^-PIPO challenged plants (Fig. 4B, top panel). In contrast, no fluorescence was observed in the upper uninoculated leaves of wild-type WMV-GFP challenged PI 414723 plants, and cell death was observed in the inoculated leaves (Fig. 4B, top panel). Immunoblot analysis showed that WMV CP accumulated in the upper uninoculated leaves of WMV-GFP-P3N^L35A^-PIPO and WMV-GFP-P3N^I43A^-PIPO infected PI 414723 plants but not in WMV-GFP, WMV-GFP-P3N^L38A^-PIPO and WMV-GFP-P3N^P41A^-PIPO challenged plants (Fig. 4C, top panel). To confirm this result, we used purified virus particles to mechanically inoculate PI 414723 plants. Wild-type WMV-GFP and above mutants were first inoculated into the susceptible YJM plants by agroinfiltration. At 20 dpai, green fluorescence and accumulation of CP were individually detected in the upper uninoculated leaves of WMV-GFP, WMV-GFP-P3N^L35A^-PIPO, and WMV-GFP-P3N^I43A^-PIPO infected YJM plants, but they were not detected in that of WMV-GFP-P3N^L38A^-PIPO and WMV-GFP-P3N^P41A^-PIPO challenged plants (Fig. 4B and C, middle panel), suggesting that the L38A and P41A mutations may affect the movement of WMV. Then, virus particles were extracted from the systemically infected YJM plants and rubbed onto the surface of PI 414723 leaves. At 20 dpmi, green fluorescence and accumulation of CP were detected in the upper uninoculated leaves of WMV-GFP-P3N^L35A^-PIPO and WMV-GFP-P3N^I43A^-PIPO infected PI 414723 plants, but they were not detected in that of WMV-GFP challenged plants (Fig. 4B and C, bottom panel). We separately extracted total RNA from WMV-GFP-P3N^L35A^-PIPO and WMV-GFP-P3N^I43A^-PIPO systemically infected PI 414723 leaves at 20 dpmi. The P3N-PIPO-encoding region of the corresponding progeny viruses was amplified by reverse transcription polymerase chain reaction and subject to sequencing. Results showed that the introduced mutations were stable in the progeny viruses of mutants (Fig. S1I). These results demonstrate that the residues L35 and I43 in the P3N domain of P3N-PIPO are crucial for WMV breaking the *Wmr* resistance.

### A few WMV isolates possess an I43V mutation that enables WMV to break the *Wmr* resistance

**Fig 5:**
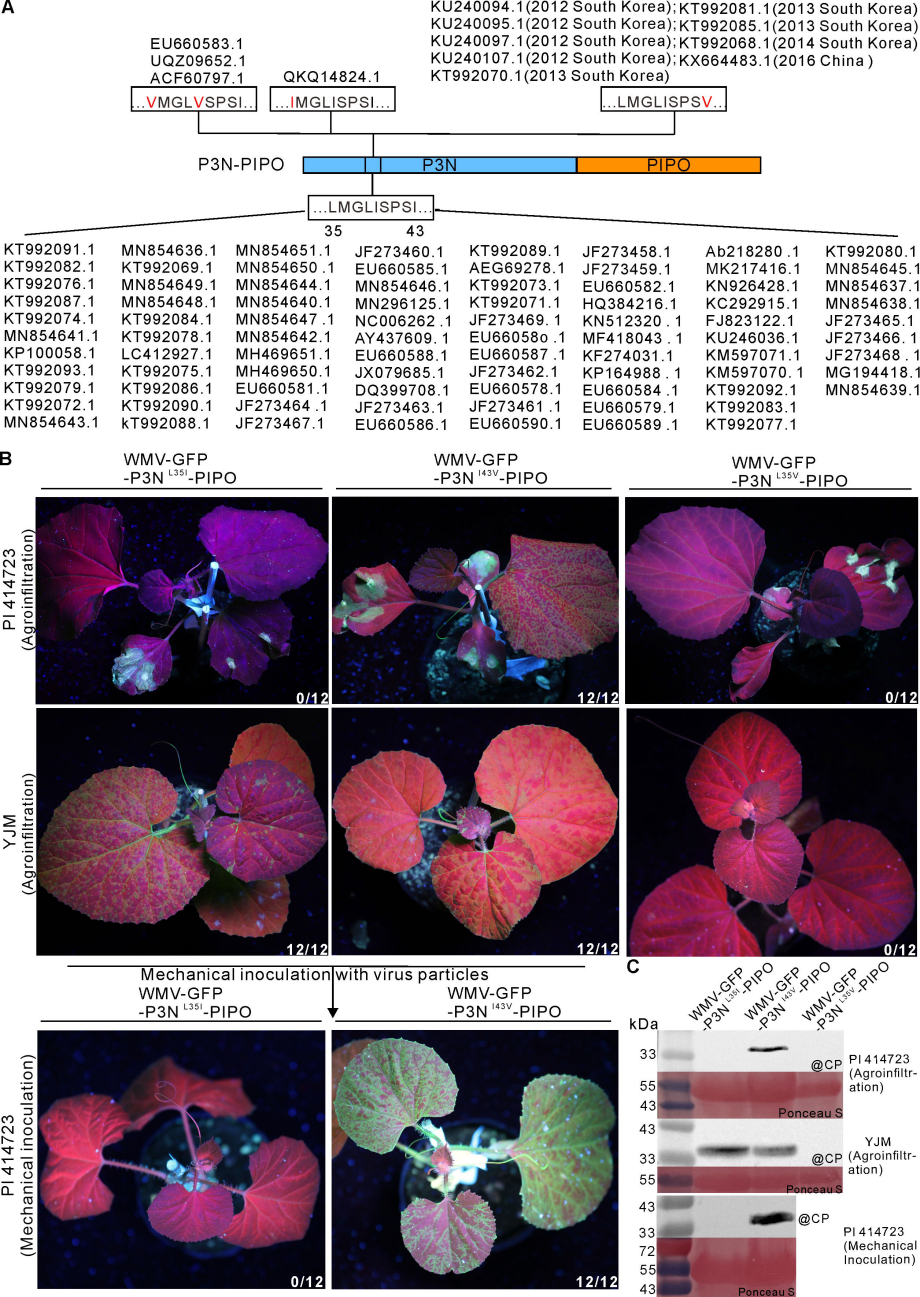
A few naturally occurring WMV isolates possess an I43V mutation that enables WMV to overcome *Wmr* resistance. (**A**) Analysis of the motif "L^35^MGLISPSI^43^" of WMV isolates sequences deposited in the NCBI database. The variant amino acid residues are indicated in red. (**B**) Phenotypes of *C. melo* plants individually inoculated with WMV-GFP-P3N^L35I^-PIPO, WMV-GFP-P3N^I43V^-PIPO, and WMV-GFP-P3N^L35V^-PIPO under UV illumination. The first panel displays the phenotypes of PI 414723 plants infiltrated with *Agrobacterium* carrying the indicated infectious clone at 10 dpai. The second panel exhibits the phenotypes of YJM plants infiltrated with *Agrobacterium* carrying the indicated infectious clone at 20 dpai. The third panel shows the phenotypes of PI 414723 plants mechanically inoculated with purified virus particles extracted from WMV-GFP-P3N^L35I^-PIPO or WMV-GFP-P3N^I43V^-PIPO infected YJM plants at 20 dpmi. The boxes indicate the inoculated leaves. The fraction represents the number of plants exhibiting fluorescence out of 12 agroinfiltrated plants from three independent experiments. (**C**) Western blot analysis of the coat protein accumulation of the upper uninoculated leaves in panel (B). Ponceau S staining shows protein loading of samples.

To investigate whether there are amino acid variations in residues L35, L38, P41, and I43 of WMV isolates in nature, we blasted the motif “L^35^MGLISPSI^43^” containing these residues in the NCBI database. Results showed that there are three types of this motif, including “V^35^MGLVSPSI^43^”, “I^35^MGLISPSI^43^,” and “L^35^MGLISPSV^43^” (the variant residue is underscored, Fig. 5A).

To test whether the changes of these residues enable WMV to break the *Wmr* resistance, the mutations were introduced into the infectious clone pCB301-WMV-GFP. The resultant mutants WMV-GFP-P3N^L35I^-PIPO, WMV-GFP-P3N^L35V^-PIPO, and WMV-GFP-P3N^I43V^-PIPO were inoculated into PI 414723 plants by agroinfiltration, respectively. At 10 dpai, green fluorescence and accumulation of CP were detected in the upper uninoculated leaves of WMV-GFP-P3N^I43V^-PIPO infected PI 414723 plants, but they were not detected in that of WMV-GFP-P3N^L35I^-PIPO and WMV-GFP-P3N^L35V^-PIPO challenged plants (Fig. 5B, top panel). We also used purified virus particles to mechanically inoculate PI 414723 plants. Likewise, these mutants were individually inoculated into the susceptible YJM plants by agroinfiltration. At 20 dpai, green fluorescence and accumulation of CP were individually detected in the upper uninoculated leaves of YJM plants infected with WMV-GFP, WMV-GFP-P3N^L35I^-PIPO, and WMV-GFP-P3N^I43V^-PIPO, but they were not detected in that of WMV-GFP-P3N^L35V^-PIPO challenged plants (Fig. 5B and C, middle panel), suggesting the L35V mutation may disrupt the infection of WMV isolate GN. Virus particles were then extracted from the systemically infected YJM plants and rubbed onto the surface of PI 414723 plants. At 20 dpmi, green fluorescence and accumulation of CP were detected in the upper uninoculated leaves of WMV-GFP-P3N^I43V^-PIPO infected PI 414723 plants, but they were not detected in that of WMV-GFP-P3N^L35I^-PIPO challenged plants (Fig. 5B and C, bottom panel). Sequencing analysis verified that the introduced mutations were stable in the progeny viruses of WMV-GFP-P3N^I43V^-PIPO in PI 414723 plants at 20 dpmi (Fig. S1I). These results suggest that an I43V variation in naturally occurring WMV isolates confers WMV ability to break the *Wmr* resistance in PI 414723.

## DISCUSSION

**Fig 6:**
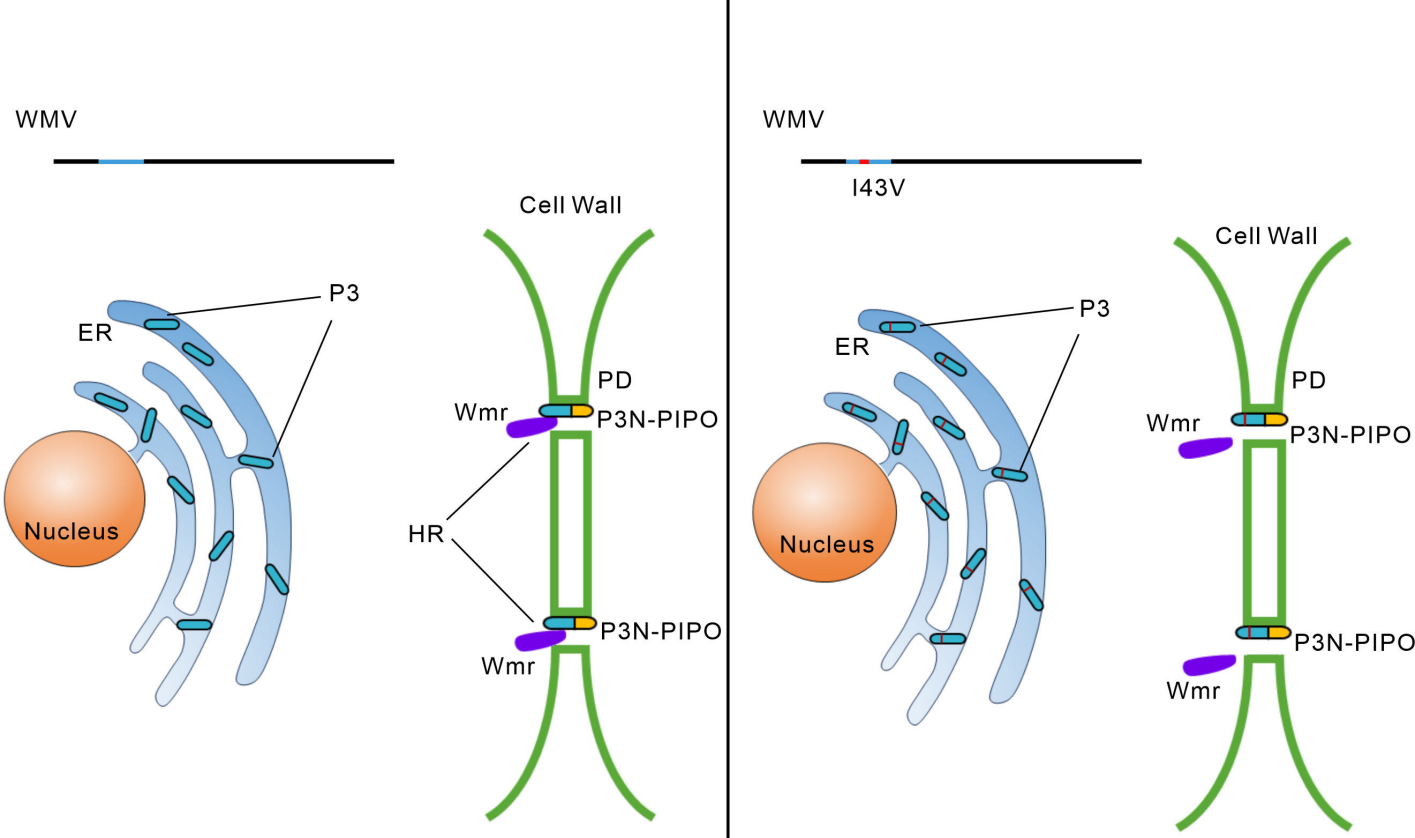
Working model describing the mechanism of the *Wmr* resistance against WMV. Wmr recognizes the P3N domain of WMV P3N-PIPO at or around PD. An I43V variation in the P3N domain from naturally occurring WMV isolates enables WMV to overcome the *Wmr* resistance.

Given the genome size of viruses is densely compact, viruses adopt a plethora of diverse translational strategies to expand their coding ability (30). Some noncanonical translational strategies, such as readthrough, frameshift, and transcriptional slippage, can produce viral proteins with common domains (7, 8). The data in this study display that two viral proteins, with common domains, target different cell compartments to be selectively recognized by an R protein (Fig. 6), which provides new insight into the interactions between R proteins and viral proteins produced by special translational strategies.

R proteins directly or indirectly recognize Avr effectors of pathogens to trigger immune responses (3). The fact that plant viruses encode viral proteins with common domains through different noncanonical translational strategies makes the interactions between R proteins and Avr effectors more complex. A canonical case is the transcriptional slippage in the genome of potyviruses, leading to the expression of P3N-PIPO embedded in the ORF of another potyviral protein P3 (12, 13, 18). Thus, both P3N-PIPO and P3 share the common P3N domain, whereas the PIPO domain is unique to P3N-PIPO. A previous study showed that P3, but not P3N-PIPO, enables SMV strain G7 to induce systemically lethal cell death in soybean with the *Rsv1* resistance gene (31, 32). TuMV P3 is also responsible for inducing cell death at the single-cell level in *Arabidopsis thaliana* Ler carrying the *TuNI* gene (33, 34). P3N-PIPO, but not P3, of ClYVV induced systemically lethal cell death in pea PI 226564. Moreover, expression of P3ΔPIPO using a WClMV-based vector did not trigger cell death, which suggests the PIPO domain plays an important role in inducing cell death (26). Distinct from previous studies, we identified that both P3N-PIPO and P3N domains trigger cell death in the melon cultivar carrying the *Wmr* gene, while P3 fails to induce cell death (Fig. 2A; Fig. S1F). We also found two amino acid residues L35 and I43 in the P3N domain are individually crucial for P3N-PIPO-triggered cell death and WMV overcoming the *Wmr* resistance (Fig. 3 and 4). From the view of genetics, *P3* can also be viewed as a genetic determinant recognized by *Wmr*. The existence of P3N-PIPO was discovered in 2008, which was much later than the time that the first potyvirus species was reported (17). Moreover, the chimeric virus derived from one resistance breaking isolate and another nonbreaking isolate was usually used for identifying virus avirulence determinants (25, 35–37). In recent years, some new viral proteins encoded by new ORFs were increasingly discovered in the genome of some plant viruses (38, 39). For example, a new ORF *P1N-PISPO*, which is produced by transcriptional slippage in a similar manner to that of *P3N-PIPO*, was identified in the *P1* cistron in sweet potato-infecting potyviruses (26, 39). Another, some small proteins that are produced by unanticipated AUG translation initiation sites and display specific subcellular localizations and virulence functions were found in two geminiviruses (38, 40). In the future, some unknown ORFs like those mentioned above may be continuously discovered as our technology advances. These hidden ORFs in viral genomes may blur the real interactions between virus avirulence genes and host resistance genes.

The subcellular localization of pathogen effectors is crucial for R proteins recognition. For example, the cognate effector from the oomycete *Hyaloperonospora arabidopsidis* Emoy2 isolate which breaks *RPP4* resistance shows cytoplasmic subcellular localization, while the effector from nonbreaking isolates of *Hpa* localizes in the nucleus (41). Here, we reveal the P3N domain, which is shared by P3 and P3N-PIPO, can trigger cell death (Fig. 2). However, P3N-PIPO, but not P3, induces cell death (Fig. 2A; Fig. S1F). Confocal microscopy analysis showed P3N-PIPO targets PD and P3N associates with PD, whereas P3 localizes in the ER (Fig. 2B). Recently, a cell wall localized NLR Rsc4-3 was demonstrated to recognize the CI protein of SMV in the apoplast (42). It can be inferred that an NLR targeting or associating with PD at the cell wall may recognize P3N-PIPO in PI 414723 (Fig. 6). Alternatively, P3N-PIPO has been shown to be released into extracellular space by vesicles in TuMV-infected leaves (43). Therefore, some receptors in the plasma membrane or extracellular space may sense P3N-PIPO and initiate the downstream signaling. Further studies on cloning the *Wmr* resistance gene will be helpful to characterize how P3N-PIPO is recognized by Wmr.

Utilization of resistant cultivars is the most effective strategy to control plant viruses and other pathogens. Avr gene-mediated phenotyping plays a significant role in rapid R gene identification and effective deployment of resistant cultivars. For example, Avr gene-based phenotyping has been established and applied to rice blast disease resistance identification in the field by using different Avr effectors (44). In this study, we identified two amino acid residues L35 and I43 in the P3N domain, which are crucial for WMV breaking the *Wmr* resistance in PI 414723 (Fig. 3 and 4). We used the motif “L^35^MGLISPSI^43^” containing these residues to scan all WMV isolates in the NCBI database and found a naturally occurring I43V variation enables WMV to completely overcome the *Wmr* resistance in PI 414723 (Fig. 5). It is worth mentioning that the number of WMV isolates containing the I43V variation increased from 2012 to 2016 in Korea and China (Fig. 5A), suggesting the resistance conferred by *Wmr* may be lost in the future. Thus, reasonable arrangement of disease-resistant varieties and the development of new resistant cultivars are particularly crucial for the prevention and control of WMV.

## MATERIALS AND METHODS

### Plant growth conditions

All varieties of cucurbit plants were germinated by soaking seeds in warm water of 25–30°C for 4–6 h, followed by growth in a dark room of 28°C. After 3 days, the seedling was moved to soil and continued to grow in greenhouse, with a photoperiod of 16/8 h (light/dark), at the temperature of 23°C with approximately 60% humidity.

### RNA extraction and reverse transcription PCR

Total RNA was extracted from PI 414723 leaf tissues using Trizol UP (TransGen Biotech) as per the manufacturer’s instruction. The RNA samples were treated with genomic DNA wiper (Vazyme), and 1 μg of total RNA was used for cDNA synthesis employing HiScript II Q RT SuperMix (Vazyme). Fragments of interest were amplified using Phanta Max Super-Fidelity DNA Polymerase (Vazyme) with primers in Table S1.

### Plasmid construction

The infectious clone of WMV was constructed as per the method described previously (45). Briefly, the full length of the WMV isolate GN was separately amplified using the primers WMV-1-1 F/WMV-1-3112 R, WMV-2-3090 F/WMV-2-7178 R as well as WMV-3-7154 F/WMV-3-10028 R, spliced using overlap extension PCR, and inserted into a pCB301 vector. A *GFP* tag was inserted between *P1* and *HC-pro*.

To construct vectors for cell death and subcellular localization assays, *P1*, *HC-Pro*, *P3*, *6K1*, *CI*, *6K2*, *NIa-Pro*, *VPg*, *NIb*, *CP*, and *P3N-PIPO* were individually PCR-amplified from pCB301-WMV-GFP using Phanta Max Super-Fidelity DNA Polymerase (Vazyme) with primers in Table S1. The resultant fragments were individually inserted into vectors of interest. *P3N-PIPO* was obtained by inserting an extra nucleotide “A” into the “GAAAAAA” region using the primer WMVP3NPIPOA F/R as per the method described previously (19, 46). The resultant plasmid was used as the template for generating P3N-PIPO mutants.

### Purification of WMV particles and mechanical inoculation

The melon leaves infected with WMV or its derivative mutants were ground in liquid nitrogen using a pestle and mortar. The ground tissue was homogenized in 0.2 M phosphate buffer (pH 8.0) containing 0.01 M EDTA and 0.15% β-mercaptoethanol for 20 min at 4°C, followed by centrifugation at 8,000 × *g* for 20 min. The resultant supernatant was mixed with polyethylene glycol 6000 (40 g/L), 1% Triton X-100, and 0.2 M NaCl. The mixture was stirred for 3 h at 4°C, followed by centrifugation at 8,000 × *g* for 20 min. The resultant pellet was resuspended overnight at 4°C in 0.2 M phosphate buffer (pH 8.0) containing 1% Triton X-100. The obtained pellet was resuspended in a 0.2-M phosphate buffer (pH 8.0) with 1% Triton X-100 and left overnight at 4°C. The solution was centrifuged at 8,000 × *g* for 20 min, and the obtained supernatant was centrifuged at 100,000 × *g* for 1 h at 4°C. The resultant pellet was resuspended overnight in 0.05 M phosphate buffer (pH 8.0) at 4°C. After a low-speed centrifugation, insoluble materials were removed. The supernatant containing purified virus particles was rubbed onto the surface of the leaves.

### *Agrobacterium*-mediated inoculation and transient protein expression

The plasmids of interest were transformed into *Agrobacterium tumefaciens* strain AGL1. *Agrobacterium* carrying the corresponding construct was suspended in an induction buffer [10 mM MgCl_2_, 10 mM 2-(*N*-morpholino) ethanesulfonic acid, and 0.15 mM acetosyringone]. The optical density at 600 nm (OD_600_) of the suspension was adjusted to 0.5 and incubated for 2–4 h at 28°C. Each suspension was infiltrated into melon leaves with a needle-free syringe.

### Western blotting

The melon leaves individually expressing proteins of interest were collected at 3 dpai, ground in liquid nitrogen, and extracted with a protein extraction buffer (10% glycerol, 25 mM Tris-HCl pH 7.5, 1 mM EDTA, 150 mM NaCl, 10 mM DL-dithiothreitol (DTT), 1× protease inhibitor cocktail, 0.5% NP-40) in a ratio of 1:1.5. For melon leaves transiently expressing proteins of interest, total protein was enriched by MonoRab Anti-DYKDDDDK Affinity Resin (GenScript). The enriched or total proteins were separated by SDS-PAGE and transferred into a nitrocellulose membrane. The membranes were incubated with THE DYKDDDDK Tag Antibody (HRP) (GenScript) or WMV CP antibody prepared in rabbit and labeled with HRP, followed by a treatment using SuperSignal West Dura substrate (Thermo Fisher Scientific), and visualized under a chemiluminescence imaging system.

### Confocal microscopy

At 3 dpai, the leaf patches with a size of about 0.5 × 0.5 cm were cut, soaked in sterilized water, covered with a glass slide, and observed under the Carl Zeiss LSM800 confocal microscope. For plasmolysis, the leaves were soaked in a 4% NaCl solution for 10–15 min before observation. The excitation wavelength of GFP was 488 nm, and the emission wavelength was 500–530 nm; the excitation wavelength of mRuby was 561 nm, and its emission wavelength was 590–630 nm.

## Data Availability

The complete genome sequence of WMV isolate GN has been deposited in GenBank under accession number OQ454911.1.

## SUPPLEMENTAL MATERIAL

Supplemental material jvi.00507-24-s0001.docx Fig. S1; Table S1.

ASM does not own the copyrights to Supplemental Material that may be linked to, or accessed through, an article. The authors have granted ASM a non-exclusive, world-wide license to publish the Supplemental Material files. Please contact the corresponding author directly for reuse.
