# Supplementary material for: P3N-PIPO but not P3 is the avirulence determinant in melon carrying the Wmr resistance against watermelon mosaic virus, although they contain a common genetic determinant
Source: J Virol. 2024 May 22;98(6):e00507-24. doi: 10.1128/jvi.00507-24 (PMC11237411; doi:10.1128/jvi.00507-24)
Supplement: Supplemental material — Fig. S1; Table S1. [file jvi.00507-24-s0001.docx]

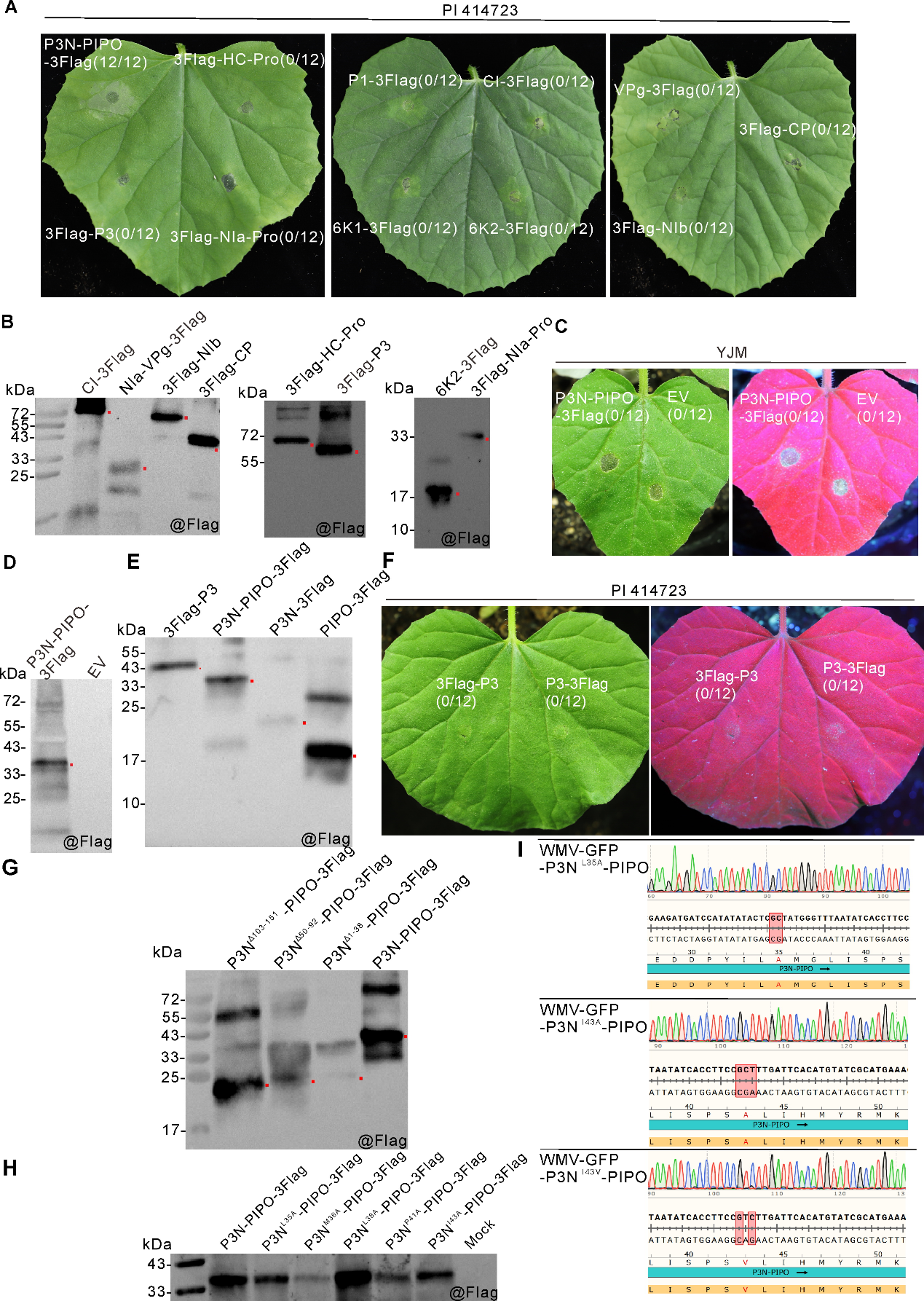


**FIG S1** (A) Phenotypes of PI 414723 leaf patches individually expressing N-terminally or C-terminally 3×Flag-tagged WMV proteins at three dpai. The fraction represents the number of leaf patches exhibiting cell death out of twelve agroinfiltrated patches from three independent experiments. (B, D, E, G, H) Western blot analysis of the expression of target proteins. Total proteins were extracted from agroinfiltrated leaves and enriched by Anti-DYKDDDDK Affinity Resin. Asterisks represent the target bands. (C) Phenotypes of YJM leaves individually expressing P3N-PIPO-3Flag and empty vector (EV) at three dpai. (F) Phenotypes of PI 414723 individually expressing 3Flag-P3 and P3-3Flag at three dpai. (I) Sequencing analysis of the progeny viruses of WMV-GFP-P3N^L35A^-PIPO, WMV-GFP-P3N^I43A^-PIPO and WMV-GFP-P3N^I43V^-PIPO in PI 414723 plants at 20 days post mechanical inoculation.

**Table S1 Primers used in this study**

| Name | Sequence（5' - 3'） |
| --- | --- |
| WMV-2916 F | CTCCTTATGGGTTTRATHTCACC |
| WMV-3511 R | GTCCAAGCYTGMTCACACTTTC |
| WMV-7004 F | CTTACGCGCAACAGYYTCAG |
| WMV-7297 R | CTAAGTTTAAAGAGCCCCAYGCR |
| WMV-1-1 F | AAATTAAAACTACTCATAAAGACATCAAAC |
| WMV-1-3112 R | GAGGACAATCTTCCAAAACATCC |
| WMV-2-3090 F | GGATGTTTTGGAAGATTGTCCTC |
| WMV-2-7178 R | CTGTTTCAAATCCATCAGTGAATGG |
| WMV-3-7154 F | CCATTCACTGATGGATTTGAAACAG |
| WMV-3-10028 R | AGGACAACAAACATTACCGTACC |
| I WMV-3-PCB301 R | TTTTTTTTTTTTTTTTTTTTTTTAGGACAACAAACATTACCGTACC |
| WMV GN-PCB301RZ F | AAAAAAAAAAAAAAAAGTCTGTACTTATATCAGTACACTGACG |
| WMV GN-PCB301 R | TGAGTAGTTTTAATTTCCTCTCCAAATGAAATGAACTTCC |
| V WMV GN(HC) F | ATAGTGTTGAATATCCCCTATCTCTCTGAA |
| V WMV GN(P1) R | ATAGTGTTGAATATCCCCTATCTCTCTGAA |
| I WMV GN-GFP F | GATATTCAACACTATTCCCACATGAGTAAAGGAGAAGAACTTTTCACTG |
| P1-3Flag F | CTGCCCAAATTCGCGATGATGGCAACAATTATGTTTGGAGATT |
| P1-3Flag R | TGAACCGCCTCCACCATAGTGTTGAATATCCCCTATCTCTC |
| 3Flag-HC-Pro F | GGTGGAGGCGGTTCATCCCACACCCCAGAGGTTCA |
| 3Flag-HC-Pro R | ATGAAACCAGAGTTAACCAACCCTGTAGAATTTCATTTCACTG |
| 3Flag-P3 F | GGTGGAGGCGGTTCAGGTGAAGTGCAACAGAGAATAAA |
| 3Flag-P3 R | ATGAAACCAGAGTTATTGTGCAGATACATCTTCCGATT |
| 6K1-3Flag F | CTGCCCAAATTCGCGATGGCCAAGACAGCCACGCAATT |
| 6K1-3Flag R | TGAACCGCCTCCACCTTGCACTTTGACTTCCTCACCCATTG |
| CI-3Flag F | CTGCCCAAATTCGCGATGAGTTTAGACGAAATTCAGAACATTGATG |
| CI-3Flag R | TGAACCGCCTCCACCCTGCAACTGCACTGCATTAAG |
| 6K2-3Flag F | CTGCCCAAATTCGCGATGAGCAAACATGAGGTTAGCAAG |
| 6K2-3Flag R | TGAACCGCCTCCACCTTGGGTTGTGACTGGTTCAC |
| VPg-3Flag F | CTGCCCAAATTCGCGATGGGAAAGAAGAGACAGATACAGAAG |
| VPg-3Flag R | TGAACCGCCTCCACCCTCTACCTCCACTCTCTCTTTG |
| 3Flag-NIa F | GGTGGAGGCGGTTCATCTAGCAAGTCTGTGTATAAGGG |
| 3Flag-NIa R | AATGAAACCAGAGTTACTGCACTGCTACTGTGTTG |
| 3Flag-NIb F | GGTGGAGGCGGTTCAAGCAGAAAGGAAAGATGGGTTCTG |
| 3Flag-NIb R | ATGAAACCAGAGTTATTGCAGAGACACTGACTCGC |
| CP-3Flag F | GGTGGAGGCGGTTCATCAGGAAAAGAAAAAGAAACAGTGG |
| CP-3Flag R  CmPDLP1-mRuby F  CmPDLP1-mRuby R | ATGAAACCAGAGTTATTACTGCGGTGGACCCATAC  CTGCCCAAATTCGCGATGGCTCCTTCTCTTCACAAC  TGAACCGCCTCCACCACCTCCATGTTTACTTTTTTTCTTCA |
| WMVP3NPIPOA F | GCATGAAAAAAATCTATGTAGATCGTTTAAAACAGGAATGG |
| WMVP3NPIPOA R | ATAGATTTTTTTCATGCGTCACGTACAGTTGATCGTTTATATCC |
| P3N-PIPO-3Flag F | CTGCCCAAATTCGCGATGATGGGTGAAGTGCAACAGAGAATAAAG |
| P3N-PIPO-3Flag R | TGAACCGCCTCCACCCACTTTCGGAAAAATGTATTCCTTACGTTTTT |
| P3N^Δ103-151^-PIPO-3Flag F | GCTTATGGAAAAAAATCTATGTAGATCGTTTAAAACAGGAATGGCAC |
| P3N^Δ103-151^-PIPO-3Flag R | TTTTTTTCCATAAGCTTTTGAGAAGTCCCTGCAATTATATCAAG |
| P3N^Δ50-92^-PIPO-3Flag F | TGTATCGCATAATTGCAGGGACTTCTCAAAAGCTTATGG |
| P3N^Δ50-92^-PIPO-3Flag R | GCAATTATGCGATACATGTGAATCAATATGGAAGGTGATAT |
| P3N^Δ1-38^-PIPO-3Flag F | TCGCGATGATATCACCTTCCATATTGATTCACATGTATCGC |
| P3N^Δ1-38^-PIPO-3Flag R | GGTGATATCATCGCGAATTTGGGCAGAATATACAGAAG |
| P3N^L35A^-PIPO-3Flag F | ATATACTCGCTATGGGTTTAATATCACCTTCCATATTGATTCACAT |
| P3N^L35A^-PIPO-3Flag R | CCCATAGCGAGTATATATGGATCATCTTCGAGAATCTG |
| P3N^M36A^-PIPO-3Flag F | ACTCCTTGCTGGTTTAATATCACCTTCCATATTGATTCAC |
| P3N^M36A^-PIPO-3Flag R | AAACCAGCAAGGAGTATATATGGATCATCTTCGAGAATCT |
| P3N^G37A^-PIPO-3Flag F | CCTTATGGCTTTAATATCACCTTCCATATTGATTCACATGTATC |
| P3N^G37A^-PIPO-3Flag R | TGATATTAAAGCCATAAGGAGTATATATGGATCATCTTCGAGAATC |
| P3N^L38A^-PIPO-3Flag F | TATGGGTGCTATATCACCTTCCATATTGATTCACATGTATC |
| P3N^L38A^-PIPO-3Flag R | TGATATAGCACCCATAAGGAGTATATATGGATCATCTTCGA |
| P3N^I39A^-PIPO-3Flag F | GGTTTAGCTTCACCTTCCATATTGATTCACATGTATCGC |
| P3N^I39A^-PIPO-3Flag R | AAGGTGAAGCTAAACCCATAAGGAGTATATATGGATCATCTTCG |
| P3N^S40A^-PIPO-3Flag F | GTTTAATAGCTCCTTCCATATTGATTCACATGTATCGCATG |
| P3N^S40A^-PIPO-3Flag R | GGAAGGAGCTATTAAACCCATAAGGAGTATATATGGATCATCT |
| P3N^P41A^-PIPO-3Flag F | TAATATCAGCTTCCATATTGATTCACATGTATCGCATGAAAC |
| P3N^P41A^-PIPO-3Flag R | AATATGGAAGCTGATATTAAACCCATAAGGAGTATATATGGATCA |
| P3N^S42A^-PIPO-3Flag F | ATCACCTGCTATATTGATTCACATGTATCGCATGAAACA |
| P3N^S42A^-PIPO-3Flag R | TCAATATAGCAGGTGATATTAAACCCATAAGGAGTATATATGGAT |
| P3N^I43A^-PIPO-3Flag F | ACCTTCCGCTTTGATTCACATGTATCGCATGAAACATTTT |
| P3N^I43A^-PIPO-3Flag R | AATCAAAGCGGAAGGTGATATTAAACCCATAAGGAGTATATATGGA |
| P3N^L35I^-PIPO-3Flag F | ATATACTCATAATGGGTTTAATATCACCTTCCATATTGATTCACAT |
| P3N^L35I^-PIPO-3Flag R | CCCATTATGAGTATATATGGATCATCTTCGAGAATCTG |
| P3N^L35V^-PIPO-3Flag F | ATATACTCGTCATGGGTTTAATATCACCTTCCATATTGATTCACAT |
| P3N^L35V^-PIPO-3Flag R | CCCATGACGAGTATATATGGATCATCTTCGAGAATCTG |
| P3N^I43V^-PIPO-3Flag F | TCCATAGCTATTCACATGTATCGCATGAAACATTTTGAG |
| P3N^I43V^-PIPO-3Flag R | GAATCAAGACGGAAGGTGATATTAAACCCATAAGGAGTATATATGGA |
